# Obesity, diabetes mellitus, and the risk of female breast cancer in Eastern China

**DOI:** 10.1186/1477-7819-11-71

**Published:** 2013-03-16

**Authors:** Xiao-Lei Wang, Cun-Xian Jia, Li-Yuan Liu, Qiang Zhang, Yu-Yang Li, Liang Li

**Affiliations:** 1 Department of Epidemiology and Health Statistics, School of Public Health, Shandong University, 44 Wenhuaxi Road, Lixia district, Jinan, Shandong, 250012, China; 2 Breast Disease Department, the Second Hospital of Shandong University, Jinan, Shandong, 250033, China

**Keywords:** Breast cancer, Female, Obesity, Diabetes mellitus, Case–control study

## Abstract

**Background:**

This study was designed to explore the relationship between obesity, diabetes mellitus (DM), and female breast cancer in Eastern China.

**Methods:**

A 1:3 matched case–control study was carried out, comprising 123 women with breast cancer and 369 controls. All of the 492 subjects were selected from a previous epidemiological survey of 122,058 women in Eastern China.

**Results:**

There were significant differences between the case and control groups in waist circumference and body mass index (BMI), but not in waist to hip ratio or hip circumference. There was a significant difference between the two groups in BMI for post-menopausal women, and a significant difference in waist circumference for pre-menopausal women. After adjustment for other factors, BMI was still significantly associated with breast cancer (odds ratio (OR) = 1.58, 95% confidence interval (CI) 1.14 to 2.19). DM was significantly associated with breast cancer (OR = 3.35, 95% CI 1.02 to 11.01) in the univariate analysis but not in the multivariate analysis (*P* = 0.059).

**Conclusions:**

Obesity might be a risk factor for female breast cancer. We found different strengths of association for women with different menopausal status when we examined the relationship between obesity and breast cancer. The association between DM and female breast cancer should be further confirmed with larger sample sizes.

## Background

Breast cancer is one of the most prevalent malignancies in women around the world. In recent years, the morbidity and mortality of breast cancer have both been increasing, and from the late 1990s, breast cancer, has replaced cervical cancer as the most common and harmful malignant tumor in women [1]. It was reported that both the rate and absolute number of female breast cancers in China showed a rapid increase between 2000 and 2005 [2], with the number of new breast-cancer cases increasing by 38.5%, and the number of deaths from breast cancer increasing by 37.1%. Reducing the incidence of female breast cancer has become an important goal worldwide.

As living standards improve, obesity and diabetes mellitus (DM) are becoming epidemic conditions not only in developed countries, but also in less developed countries such as China. Between the 1992 and 2002, the combined prevalence of overweight and obesity increased from 20.0% to 29.9%, based on the Chinese body mass index (BMI) cut-off points, and the annual increase rate was highest in women aged 45 to 59 years old [3], which was also the age group with the highest rate of breast cancer [4]. A national survey conducted in 1994 in China showed that the prevalence of DM was 2.5% [5], whereas in 2007, the prevalence had reached 9.7% [6]. Some researchers have found an association betwen obesity or DM and the mortality rates from breast cancer [7,8], but others have found no such assocation [9]. Because of these inconsistencies regarding the association between obesity or DM and breast cancer, it is necessary to study this relationship further.

In this study, we examined the relationship between obesity and DM and breast cancer in a Chinese female population. We used 1:3 matched case–control study to explore the association between obesity and DM and female breast cancer, enrolling 123 women with breast cancer and 369 controls [10].

## Methods

### Ethics approval

This program was approved by the ethics committee of the Second Hospital of Shandong University (principal unit of this program). Informed consent was obtained from all participants in accordance with the ethical standards of the responsible committee on human research.

### Study population

All the women with breast cancer and controls were ascertained from our previous epidemiology survey in Eastern China, which was carried out using multistage stratified cluster sampling method during the period from 15 July to 15 September 2008, in Shandong, Hebei, Jiangsu, and Tianjin, in Eastern China [10]. For that survery, women aged 25 to 70 years old were interviewed. and the data of 122,058 women were obtained. All women with breast-cancer cases underwent a clinical examination, ultrasonography and screening mammography for confirmation of breast cancer.

We ascertained patients who had been living in the study areas for at least 2 years. In total, 123 patients with breast cancer were identified. Pathological data were available for 103 of these patients, while 20 further cases had been diagnosed by specialist cancer hospitals, but pathological data was not availale for these. Hence, 123 cases of breast cancer diagnosed in previous two years were selected as the case group, and 369 control subjects were selected to form a 1:3 matched case–control study. The controls were the same age (±2 years), were living in the same area for at least 2 years, and had no blood relationship with the women with breast cancer. All the controls underwent clinical examination and ultrasography to ensure they were healthy. All the cases and controls were of Han ethnicity.

### Data collection

The data collected included personal information (age, weight, height, residence, occupation, educational level, personal and family income), menstrual and reproductive history (marriage, reproductive history, breastfeeding history, and age at menarche and menopause), family history (breast cancer history: first-degree, second-degree and third-degree relatives with breast cancer), disease history (benign breast disease, gynecologic tumor, DM, hypertension, coronary heart disease and nephritis), lifestyle (smoking, drinking, physical exercise, quality-of-life satisfaction), and dietary habits.

### Quality control

In the previous study, each subject underwent a clinical examination by two physicians, each of whom had over 3 years of experience working in a breast-surgery department. Each subject completed a questionnaire through interview by one of our trained questionnaire interviewers. At the end of each day, 10% of the questionnaires were randomly inspected to check for completeness, accuracy, and standardization. Any missing data or errors were corrected the following day. After the whole investigation was completed, the questionnaires were entered twice into a computer by two different inputters blinded to the study goals, with the second person correcting any inconsistencies found in the first set of data. Detailed information is available in the previously published paper [10].

### Statistical analysis

In this study, the data were analyzed using SPSS software (version 16.0; SPSS Inc., Chicago, IL, USA). The *χ*^2^ and *P*-values were calculated to compare whether there were significant differences in frequencies and percentages of the analyzed variables between the breast-cancer case and control groups. Univariate and multivariate Cox regression models, whose effects were equal to those of conditional logistic regression models in the analysis to obtain *P*-values and odds ratios (ORs) with 95% confidence intervals (95% CIs) of the variables. The statistical significance was set at a two-sided *P*-value of 0.05.

## Results and discussion

### The demographic characteristics of breast-cancer case and control groups

The mean age (± standard deviation; SD) was 49.94 (±9.44) for the entire population (123 breast-cancer cases and 369 controls), 49.95 ± 9.48 for the cases alone, and 49.92 ± 9.37 for the controls alone. No significant differences were found between the breast-cancer case and control groups for age distribution (*χ*^*2*^ = 0.659, *P* = 0.417), residence (*χ*^*2*^ = 2.517, *P* = 0.113), or education level (*χ*^*2*^ = 0.042, *P* = 0.838). However, there were significant differences between these two groups for family history of breast cancer (*χ*^*2*^ = 3.828, *P*<0.001), total life satisfaction (*χ*^*2*^ = 15.990, *P*<0.001), current life satisfaction (*χ*^*2*^ = 12.461, *P*<0.001), and frequency of exercise (*χ*^*2*^ = 5.207, *P* = 0.022), and the *P*-value for annual family income was close to significance (*χ*^*2*^ = 3.828, *P* = 0.05) (Table 1).

**Table 1:** Demographic characteristics of breast cancer and control groups

**Characteristic**	**Cases, n (%)**	**Controls n (%)**	***χ***^***2 ***^**(df)**	***P *value**
Age				
25 to 35	5 (4.1)	14 (3.8)	0.659 (3)	0.417
36 to 49	56 (45.5)	162 (43.9)		
50 to 64	54 (43.9)	167 (45.3)		
65 to 70	8 (6.5)	26 (7.0)		
Residence				
Urban	27 (22.0)	97 (26.3)	2.517 (1)	0.113
Rural	96 (78.0)	272 (73.7)		
Education				
Elementary or low	58 (47.2)	192 (52.0)	0.042 (3)	0.838
Middle	36 (29.3)	86 (23.3)		
High	24 (19.5)	60 (16.3)		
College	5 (4.0)	31 (8.4)		
Family annual income (RMB)				
<15,000	54 (43.9)	191 (51.8)	3.828 (1)	0.050
≥15,000	69 (56.1)	178 (48.2)		
Family history of breast cancer				
Yes	10 (8.1)	7 (1.9)	16.056 (1)	0.000
No	113 (91.9)	362 (98.1)		
Total life satisfaction^a^				
<25	46 (37.4)	215 (58.3)	15.990 (1)	0.000
≥25	77 (62.6)	154 (41.7)		
Current life satisfaction^ab^				
<13	43 (35.0)	207 (56.1)	12.461 (1)	0.000
≥13	80 (65.0)	162 (43.9)		
Exercise				
Yes	13 (10.6)	72 (19.5)	5.207 (1)	0.022
No	110 (89.4)	297 (80.5)		

Abbreviations: df, degrees of freedom; RMB, renminbi.

^a^Total life satisfaction was rated with a cumulative score of 12 items, with high scores indicating low life satisfaction, and low scores indicating high life satisfaction.

^b^Current life satisfaction was rated with a cumulative score of six items, with high scores indicating low life satisfaction, and low scores indicating high life satisfaction.

### The association between indexes related to obesity and female breast cancer

Of the four common indexes of obesity, no significant differences were found between the breast-cancer case and control groups for waist to hip ratio (WHR) (*χ*^*2*^ = 0.121, *P* = 0.727) or hip circumference (HC) (*χ*^*2*^ = 1.169, *P* = 0.280), but there were significant differences in the distribution of BMI (*χ*^*2*^ = 6.603, *P* = 0.010) and waist circumference (WC) (*χ*^*2*^ = 7.255, *P* = 0.007) (Table 2).

**Table 2:** Distribution of obesity-related indexes between the female breast-cancer case and control groups

**Measurement**	**Cases, n (%)**	**Controls, n (%)**	***χ***^***2 ***^**(df)**	***P *value**
BMI				
<24.0	56 (45.5)	197 (53.4)	6.603 (2)	0.010
24.0 to 28.0	42 (34.2)	134 (36.3)		
≥28.0	25 (20.3)	38 (10.3)		
WHR				
<0.85	89 (72.4)	270 (73.2)	0.121 (1)	0.727
≥0.85	34 (27.6)	99 (26.8)		
WC				
<80	57 (46.3)	219 (59.3)	7.255 (1)	0.007
≥80	66 (53.7)	150 (40.7)		
HC				
<100	63 (51.2)	208 (56.4)	1.169 (1)	0.280
≥100	60 (48.8)	161 (43.6)		

Abbreviations: BMI. body mass index; df, degrees of freedom; HC. hip circumference; WC, waist circumference; WHR.: waist to hip ratio.

When the results were stratified by different menopausal status of the women in the breast-cancer case and control groups, there were no significant differences in BMI in the pre-menopausal group, WC in the post-menopausal group, and WHR and HC in both the pre-menopausal and post-menopausal groups. However, there were significant differences in BMI for the post-menopausal group (*χ*^*2*^ = 9.645, *P* = 0.008), and WC for the pre-menopausal group (*χ*^*2*^ = 4.701, *P* = 0.030) (Table 3).

**Table 3:** Association between obesity-related indexes and female breast cancer in patients with different menopausal status

**Measurement**	**Menopausal status**^**a**^	**Cases n (%)**	**Controls n (%)**	***χ***^***2 ***^**(df)**	***P *value**
BMI					
<24.0	0	28 (47.4)	100 (53.2)	0.752 (2)	0.687
24.0 to 28.0		24 (40.7)	71 (37.8)		
≥28.0		7 (11.9)	17 (9.0)		
<24.0	1	28 (43.8)	97 (53.6)	9.645 (2)	0.008
24.0 to 28.0		18 (28.1)	63 (34.8)		
≥28.0		18 (28.1)	21 (11.6)		
WHR					
<0.85	0	44 (74.6)	150 (79.8)	0.724 (1)	0.395
≥0.85		15 (25.4)	38 (20.2)		
<0.85	1	45 (70.3)	120 (66.3)	0.346 (1)	0.556
≥0.85		19 (29.7)	61 (33.7)		
WC					
<80	0	30 (50.8)	125 (66.5)	4.701 (1)	0.030
≥80		29 (49.2)	63 (33.5)		
<80	1	27 (42.2)	94 (51.9)	1.797 (1)	0.180
≥80		37 (57.8)	87 (48.1)		
HC					
<100	0	36 (61.0)	112 (59.6)	0.039 (1)	0.844
≥100		23 (39.0)	76 (40.4)		
<100	1	27 (42.2)	96 (53.0)	2.227 (1)	0.136
≥100		37 (57.8)	85 (47.0)		

Abbreviations: BMI, body mass index; df, degrees of freedom; HC, hip circumference; WC, waist circumference; WHR, waist to hip ratio.

^a^Menopausal status: 0, pre-menopausal status; 1, post-menopausal status.

### The association between breast cancer and physiological, reproductive, clinical, behavioral, and dietary factors

Other possible factors that might be related to breast cancer, including physiological, reproductive, clinical, behavioral, and dietary factors, were also analyzed. We did not find any significant differences between the breast-cancer case and control groups for menstrual history, including age at menarche (*χ*^*2*^ = 1.496, *P* = 0.221), menstrual pattern (*χ*^*2*^ = 2.446, *P* = 0.118), dysmenorrhea (*χ*^*2*^ = 0.382, *P* = 0.536), or menopausal status (*χ*^*2*^ = 0.643, *P* = 0.423); reproductive history, including age at first birth (*χ*^*2*^ = 1.613, *P* = 0.204), number of births (*χ*^*2*^ = 1.433, *P* = 0.231), or breastfeeding status (*χ*^*2*^ = 0.057, *P* = 0.811); or history of hypertension (*χ*^*2*^ = 1.246, *P* = 0.264), cardiovascular disease (*χ*^*2*^ = 1.712, *P* = 0.191), nephritis (*χ*^*2*^ = 0.333, *P* = 0.564), or gynecologic tumors (*χ*^*2*^ = 0.914, *P* = 0.339). However, significant differences between the case and control groups were found for number of miscarriages (*χ*^*2*^ = 8.660, *P* = 0.003), history of benign breast tumor (*χ*^*2*^ = 12.075, *P* = 0.001) and presence of DM (*χ*^*2*^ = 4.459, *P* = 0.035).

No significant differences were found for other factors including oral contraception (*χ*^*2*^ = 0.000, *P* = 0.997), inverted nipple (*χ*^*2*^ = 1.731, *P* = 0.188), nipple discharge (*χ*^*2*^ = 0.150, *P* = 0.699), orgalactophore hyperplasia (*χ*^*2*^ = 0.441, *P* = 0.507), or for dietary habits, including intake of bean products (*χ*^*2*^ = 2.578, *P* = 0.108), fresh beans (*χ*^*2*^ = 0.022 *P* = 0.883), red meat (*χ*^*2*^ = 0.010, *P* = 0.919), milk products (*χ*^*2*^ = 0.422, *P* = 0.516), corn (*χ*^*2*^ = 543, *P* = 0.461), carrots (*χ*^*2*^ = 0.319, *P* = 0.572), fried food (*χ*^*2*^ = 0.721, *P* = 0.396), vegetables or fruit (*χ*^*2*^ = 0.630, *P* = 0.427), garlic (*χ*^*2*^ = 3.316, *P* = 0.069), hams (*χ*^*2*^ = 0.763, *P* = 0.383), or pickled foods (*χ*^*2*^ = 0.583, *P* = 0.445).

### Results of univariate logistic regression analysis in the 1:3 matched case–control study

Univariate logistic regression analysis showed that female breast cancer was significantly associated with BMI, WC, DM, total life satisfaction, exercise, number of miscarriages, family history of breast cancer, and history of benign breast tumor (Table 4).

**Table 4:** Results of univariate and multivariate conditional logistic regression model analysis in 1:3 matched case–control study of 123 women with breast cancer

	**Univariate analysis**				**Multivariate analysis**			
	***P***	**OR**	**95% CI**		***P***	**OR**	**95% CI**	
Total life satisfaction	0.014	1.04	1.01	1.07	0.000	0.42	0.27	0.66
Exercise	0.025	0.48	0.25	0.91	–	–	–	–
Number of miscarriage	0.004	1.64	1.17	2.30	0.042	1.47	1.01	2.14
Family history of breast cancer	0.001	5.19	1.92	14.04	0.006	4.52	1.53	13.34
History of benign breast tumor	0.002	4.62	1.79	11.91	0.003	4.66	1.66	13.10
BMI	0.011	1.48	1.09	2.00	–	–	–	–
Diabetes mellitus	0.047	3.35	1.02	11.01	0.059	3.49	0.95	12.81
Waist circumference	0.008	1.84	1.18	2.87	–	–	–	–

Abbreviations: BMI, body mass index; CI, confidence interval; OR, odds ratio.

### Results of multivariate logistic regression analysis in the 1:3 matched case–control study

Multivariate logistic regression analysis using the forward method was carried out on the significant variables found by univariate logistic regression analysis. The results indicated that there were four factors significantly associated with female breast cancer, which were family history of breast cancer (OR = 4.52, 95% CI 1.53 to 13.34), history of benign breast tumor (OR = 4.66, 95% CI 1.66 to 13.10), BMI (OR = 1.58, 95% CI 1.14 to 2.19), number of miscarriages (OR = 1.47, 95% CI 1.01 to 2.14), and total life satisfaction (OR = 0.42, 95% CI 0.27 to 0.66) (Table 4). DM was not significantly associated with female breast cancer in the multivariate analysis (Table 4).

### Discussion

In this study, the major findings were as follows 1) BMI, an index of obesity, was significantly associated with female breast cancer. When the data were stratified according to menopausal status, BMI was significantly associated with breast cancer in the post-menopausal group, whereas WC was significantly associated with breast cancer in the pre-menopausal group. 2) DM was significantly associated with female breast cancer in the univariate analysis; however, no significant difference was found in the multivariate analysis (*P* = 0.059). 3) Family history of breast cancer, history of benign breast tumor, number of miscarriages, and total life satisfaction were also important factors related to breast cancer.

As economies have developed, living conditions have improved. However, the structure of the diet has also changed, with more high-fat food being eaten, while at the same time, the need for physical strength in work has diminished, and these factors have resulted in a rising level of obesity with consequent morbidity and mortality. Usually, BMI is used to evaluate generalized obesity, whereas WHR and WC are used to evaluate central obesity. Many studies have indicated that BMI is an important index for evaluating the degree of obesity and the association between obesity and breast cancer [10], but confirmatory studies performed in Asian populations are lacking. The results of a census carried out on women in a community in Shanghai indicated that a BMI of ≥25 is a risk factor for breast cancer in pre-menopausal women, and a BMI of ≥30 is a risk factor for breast cancer for post-menopausal women, BMI ≥30 is a risk factor [11]. Many studies have concluded that obesity may increase the risk of breast cancer in post-menopausal women, but no association has been identified for pre-menopausal women [8,12,13]. Supporting these findings, we found that in the current study, BMI was significantly associated with female breast cancer, and may increase the risk of breast cancer in post-menopausal women.

The association between breast cancer and central obesity is inconsistent. Some studies have indicated that central obesity is a risk factor for female breast cancer [14], whereas others have found no association [15]. In the current study, we found a significant difference between the breast-cancer cases and control groups for WC in the pre-menopausal group, whereas no association was found between WHR and breast cancer. These results indicate that WC may be a risk factor for pre-menopausal women and thus should receive more attention to in breast-cancer prevention.

A cohort study started in 1976 in the USA, enrolling 116,488 female nurses as research subjects [16]. The results of the 20-year follow-up showed that women with type 2 DM had a 17% higher risk of developing breast cancer than women without type 2 DM. Another cohort study also indicated DM is significantly associated with breast cancer [17]. In the current study, we found that DM was significantly associated with female breast cancer in the univariate analysis and showed a trend towards association (*P* = 0.059) in the multivariate analysis. This indicates that DM may be an independent factor related to female breast cancer in Eastern China. Because of the huge population of China, even a low increase in the rate of DM may result in a large number of patients, thus, in both public health and clinical practice, there should be more attention paid to the role of DM in breast-cancer prevention.

Both DM and breast cancer have a number of risk factors in common, including age and smoking [18], and obesity is also an important risk factor for type 2 DM [19]. Obesity is associated with diabetes progression, and if insulin resistance is related to breast cancer, then obesity might be a part of the interplay between insulin resistance, DM, and breast cancer [20]. Owing to their effects on adipocytokines and inflammatory mediators, both obesity and type 2 diabetes have been shown to contribute to the increasing breast-cancer risk in pre-menopausal women [21].

A family history of breast cancer and personal benign breast disease are regarded as risk factors for breast cancer [22,23], and we also found in the current study that a family history of breast cancer and personal benign breast disease were also significantly associated with breast cancer.

There is currently no consensus regarding the effect of number of miscarriages on breast cancer [24,25]. In the current study, both the univariate (OR = 1.64, 95% CI 1.17 to 2.30) and multivariate (OR = 1.47, 95% CI 1.01 to 2.14) logistic regression analyses indicated that the number of miscarriages was significantly associated with female breast cancer.

The multivariate analysis also indicated that higher life satisfaction was protective against breast cancer (OR = 0.42, 95% CI 0.27 to 0.66). As life satisfaction is a comprehensive response of a personal or family conditions, these results indicate that actively improving the life satisfaction levels of a population might be of benefit for breast cancer prevention.

The amount of exercise was significantly different between the breast-cancer case and control groups, and the result of the univariate logistic regression analysis indicates that exercise might be protective against breast cancer. A previous population-based study found that recreational physical activity provided benefit for reducing breast-cancer risk [26], and physical activity might offer one modifiable lifestyle characteristic that may substantially reduce a woman’s lifetime risk of breast cancer [27].

Several limitations to the present study should be noted here. First, all the breast-cancer cases and controls were women aged 25 to 70 years old, who were all of Han ethnicity. Because China is a multi-ethnic country, our results cannot be extended to the entire Chinese female population, because of differences in age and ethnicity. Second, the disease history of participants, including DM and hypertension, was collected by subject self-reports, thus some women with DM and hypertension may have been missed. Third, age is associated with breast cancer, but in our study, age was the matching variable, thus the association between age and breast cancer could not be analyzed. Fourth, one previous study reported that the incidence of breast cancer was different in diabetes patients depending on whether or not they were taking metformin [28]; however, in the present study, the information on drug intake of patients with diabetes was unclear. Thus, this issue needs to be targeted in future studies.

## Conclusions

Obesity is a risk factor for breast cancer, with specific risk factors being BMI in post-menopausal women and WC in pre-menopausal women. There also seems to be a trend for the association between DM and female breast cancer in China. For both public health and clinical practice, obesity and DM should be considered risk factors for female breast cancer, along with the existing factors, such as family history of breast cancer, history of benign breast disease, and number of miscarriages.

## Competing interests

The authors declare that they have no competing interests.

## Authors’ contributions

JCX carried out the study conception and design and helped to draft the manuscript. WXL performed the statistical analysis and manuscript writing. LLY participated in the design of the study and was responsible for data collection and database setting. ZQ and LL participated in the design and coordination of the study and helped to perform the investigations. LYY was responsible for data collection and quality control. All authors read and approved the final manuscript.
